# Investigating an abnormal hump phenomenon in top gate a-InGaZnO thin-film transistors due to mobile sodium diffusion

**DOI:** 10.1038/s41598-023-40664-x

**Published:** 2023-08-22

**Authors:** So Hee Park, Min Young Kim, Hyeong Wook Kim, Changyong Oh, Hyeong Keun Lee, Bo Sung Kim

**Affiliations:** 1 Department of Applied Physics, Korea University, Sejong, 30019 Republic of Korea; 2 E·ICT-Culture·Sports Track, Korea University, Sejong, 30019 Republic of Korea; 3 Division of Display and Semiconductor Physics, Korea University, Sejong, 30019 Republic of Korea

**Keywords:** Electronic devices, Applied physics

## Abstract

Top gate a-InGaZnO (IGZO) thin-film transistors (TFTs) annealed at high temperature show excellent initial current–voltage (I–V) characteristics. However, when they are exposed to positive gate bias for a long time, hump can occur in the subthreshold region. This abnormal hump is accelerated at a higher positive gate voltage and mitigate by a negative gate voltage. While the strength of the hump is irrelevant to a change in channel width, it relies significantly on channel length. This phenomenon might be due to mobile Na ions diffused from a glass substrate migrating toward the back and edge side of the IGZO semiconductor by a vertical gate electric field. When a layer of Al_2_O_3_ is formed between the IGZO semiconductor and the glass substrate, the hump phenomenon could be successfully solved by serving as a barrier for Na ions moving into the IGZO.

## Introduction

Amorphous InGaZnO (a-IGZO) thin-film transistors (TFTs) have attracted much attention as switching or driving devices of TFT backplanes for applications of flexible, transparent, and large-area displays^1–3^. Due to their advantages such as large-current driving capability, low leakage current, low-temperature process, and superior large area manufacturing uniformity, a-IGZO TFTs are promising candidates that can meet high-performance requirements of advanced active-matrix organic light-emitting diodes (AMOLEDs) such as high resolution, high frame rate driving, and low power consumption^4–6^. However, various types of defects in the bulk or channel interface of an a-IGZO semiconductor can cause problems with long-term electrical stability of the TFT device. Many studies have determined effects of defect states in a-IGZO on electrical properties and reliability of a-IGZO TFT according to external stresses such as voltage, temperature, illumination, and mechanical force^7–10^. When observing changes in threshold voltage (V_th_) of a-IGZO TFTs against various electrical stresses such as positive gate voltage bias, negative gate voltage bias, and gate bias stress with illumination, a hump might occur in the subthreshold region of current–voltage (I–V) characteristics^11–13^. These bias stress-induced hump phenomena can occur due to formation of a parasitic current path by creating unintentional parallel transistors in local active regions of a-IGZO TFTs^14–18^. They can eventually cause changes of subthreshold slope or on-set voltage in I–V characteristics of TFTs, resulting in high-pixel defects and bad pixel-uniformity on OLED display panels. Hump phenomena reported in a-IGZO TFTs can be caused by several factors such as back-channel conduction due to migration of various ionized defects inside IGZO semiconductors, charge trapping in the edge region of IGZO active islands, and impact ionization due to a high drain current^15–20^. Therefore, when a hump occurs in a TFT device, it is important to analyze the cause and present a solution for this phenomenon known to deteriorate display images.

In this paper, we investigated a time-delayed hump phenomenon that occurred under a positive gate bias condition of high voltage for a long time in top gate a-IGZO TFTs with excellent I–V characteristics. Changes in the hump for stress time were observed according to different channel widths (W) and lengths (L) of TFTs. In addition, the cause and mechanism of hump generation were identified through various electrical stress measurements and spectrometry analyses of ions in a-IGZO films. It was found that some Na ions diffused into the a-IGZO film from the glass substrate could cause a hump in I–V characteristics. An improvement method was proposed to remove the hump from the a-IGZO TFT.

## Methods

A borosilicate glass (Schott, Borofloat®, 0.7 mm, UK) was used as a substrate to fabricate a IGZO TFT device. Indium Tin Oxide (ITO; 20 nm) was deposited and patterned to form source and drain (S/D) electrodes on top of the glass substrate. IGZO (In: Ga: Zn = 1: 1: 1 atomic%, 30 nm) was then deposited with a radio frequency (RF)-magnetron sputtering system. An RF power source (13.56 MHz, 100 W) was employed for depositing IGZO layers. During deposition, the chamber working pressure was set to be 0.67 Pa with argon (Ar): oxygen gas (O_2_) flow rate ratio of 35: 5 standard cubic centimeters per minute at room temperature. IGZO films were photo-patterned and wet etched with a buffered oxide etchant (200:1, Mattech Resource). Al_2_O_3_ as a gate insulator layer was deposited by plasma enhanced atomic layer deposition (PEALD) using trimethyl aluminum (TMA) as a metal source and O_2_ as a reactant. Plasma power was set to be 100 W at 250 °C. One cycle of atomic layer deposition (ALD) was performed with the following sequence: TMA source injection (0.5 s)–argon (Ar) purge (3.0 s)–O_2_ injection (1.0 s)–RF-plasma (0.5 s)–Ar purge (5 s). The thickness of Al_2_O_3_ layers was set to be 150 nm. After S/D contact holes were formed on the Al_2_O_3_ dielectric layer by photolithography, Mo (50 nm) gate electrode was sputter-deposited and patterned to afford IGZO TFTs with a top-gate staggered configuration. These IGZO TFTs were post-annealed at 350 °C for 1 h. Meanwhile, an IGZO TFT with a barrier layer on the glass substrate was fabricated as a comparative TFT device. Before depositing ITO S/D, an Al_2_O_3_ barrier layer (50 nm) was formed on the substrate using PEALD under the same deposition conditions (250 °C, 100 W) as a gate insulator. All other TFT processes except for the barrier layer were carried out in the same way. I–V characteristics of a-IGZO TFTs were then measured with gate voltage (V_g_) sweep of − 20 V to + 20 V at a constant drain voltage (V_d_) of + 10 V using a semiconductor device analyzer (B1500A, Agilent) at room temperature under dark air ambient condition. Time of flight-secondary ion mass spectrometry (TOF–SIMS; M6, IONTOF GmbH) was conducted to determine the amount of each component such as In, Ga, Zn, H, OH, and Cl ions in IGZO films. A cesium (Cs^+^) primary ion beam was used at a current of 120 nA and a raster size of 200 × 200 mm^2^. Chemical states of oxygen atoms in Al_2_O_3_/IGZO/glass stack and Al_2_O_3_/IGZO/Al_2_O_3_/glass stack were examined by X-ray photoelectron spectroscopy (K-Alpha^+^, Thermo Fisher Scientific).

## Results and discussion

### Electrical characteristics and bias stress stability of top gate a-IGZO TFTs

**Figure 1:**
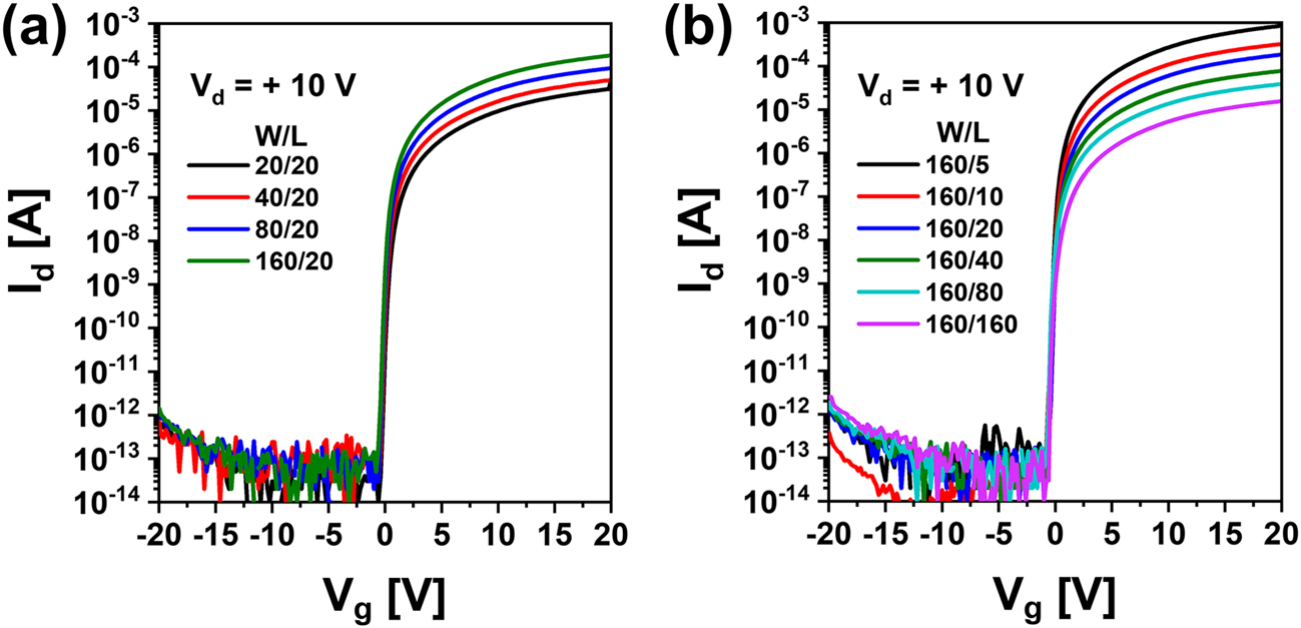
Electrical characteristics of top gate a-IGZO TFTs according to (**a**) different channel widths of 20, 40, 80, and 160 μm with a fixed length of 160 μm and (**b**) different channel lengths of 5, 10, 20, 40, 80, 160 μm with a fixed width of 160 μm after annealing at 350 °C for 1 h**.**

**Figure 2:**
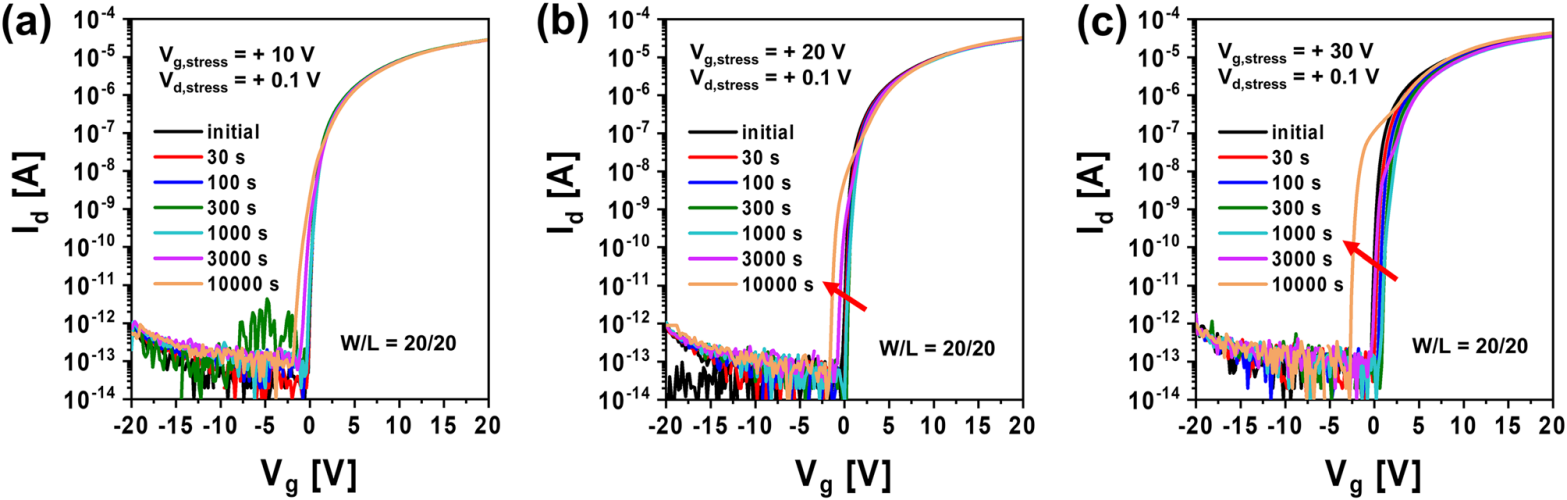
I–V characteristics for a-IGZO TFTs under positive gate bias stress of (**a**) V_g_ =  + 10 V, (**b**) V_g_ =  + 20 V and (**c**) V_g_ =  + 30 V.

**Figure 3:**
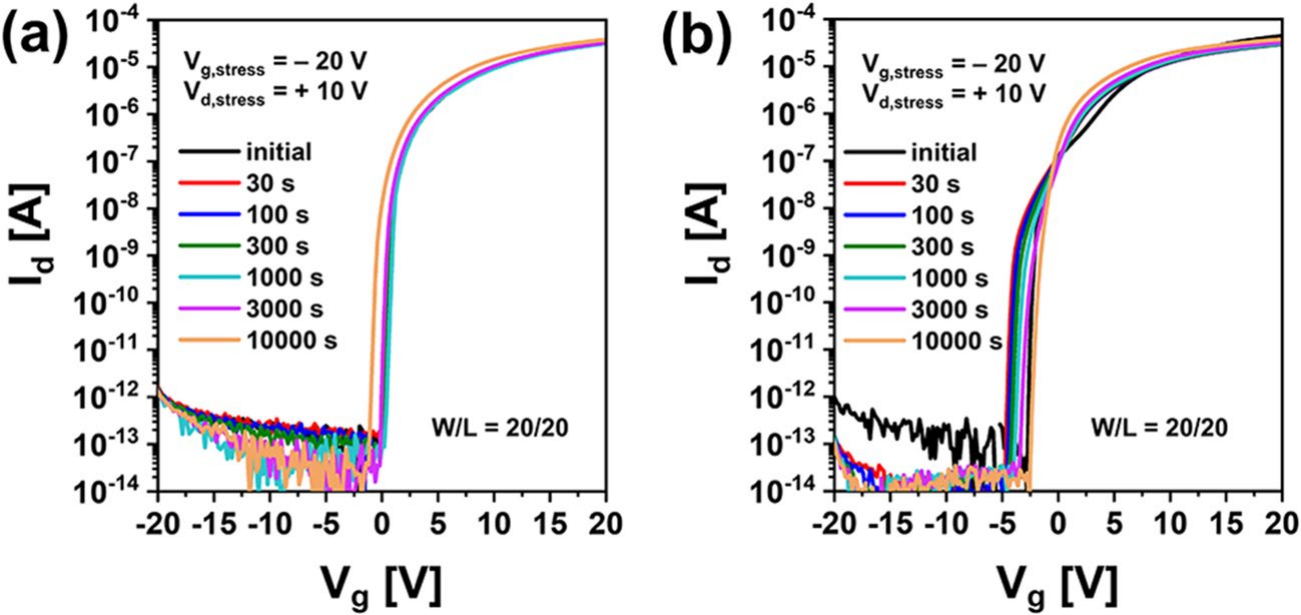
I–V characteristics for a-IGZO TFTs according to stress time under negative gate bias condition of V_g_ = − 20 V and a drain voltage of + 10 V (**a**) from an initial state of a pristine TFT without stress and (**b**) those from the hump state after stress under a positive gate bias for 10,000 s.

**Figure 4:**
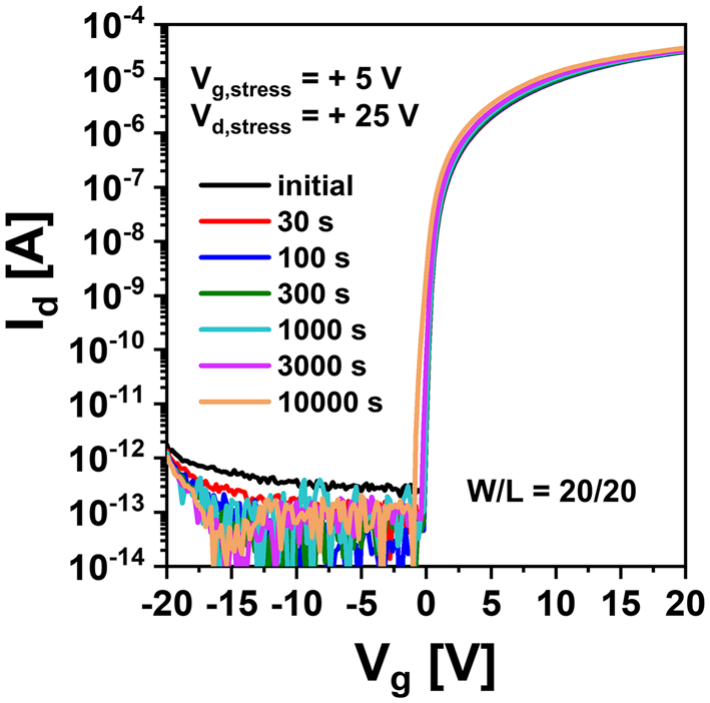
I–V characteristics of a-IGZO TFTs under high drain bias stress of V_d_ =  + 25 V and gate voltage of + 5 V (V_dg_ =  + 20 V).

Figure 1a,b show I–V characteristics of top gate a-IGZO TFTs with different channel widths (*W* = 20, 40, 80, 160 μm with a fixed *L* of 20 μm) and different channel lengths (*L* = 5, 10, 20, 40, 80, 160 μm with a fixed W of 160 μm) after annealing at 350 °C for 1 h, respectively. These a-IGZO TFTs exhibited excellent electrical performances and uniformity with field effect mobility of 7.3 ± 0.92 cm^2^V^−1^ s^−1^, V_th_ of 0.25 ± 0.33 V, subthreshold slope (SS), and 112 ± 44 mVdec^−1^ in the above-described range of channel width by length (W/L). To examine electrical reliabilities of these a-IGZO TFTs, changes in their I–V curves were observed after they were stressed under positive gate bias condition applying gate voltages of + 10 V, + 20 V, and + 30 V at a fixed drain voltage of + 0.1 V for 10,000 s, respectively. As the gate voltage of TFTs increased, the V_th_ of transfer curves shifted more to the positive direction as shown in Fig. 2. This could be attributed to an increase in charge trapping at the channel interface of the IGZO semiconductor by a prolonged positive gate bias^21,22^. Meanwhile, when the stress time reached 10,000 s under positive gate bias stress (PBS), a weak hump appeared in subthreshold region of the I–V curve (Fig. 2a). As shown in Fig. 2b,c, considering that the hump was significantly increased with increasing gate voltage to + 20 V and + 30 V, it was found that this hump phenomenon was generated obviously by a gate bias stress. We noted the occurrence of this abnormal hump at long-term direct current (DC) gate bias stress. To determine whether the same hump would appear in negative gate field, a sustained negative gate bias stress (NBS) to the a-IGZO TFT was applied under the condition of a gate voltage of − 20 V and a drain voltage of + 10 V for 10,000 s. As shown in Fig. 3a, there was little change in the I–V curve up to 3,000 s at NBS. Although the V_th_ shifted slightly negatively (ΔV_th_ = – 0.98 V) after 10,000 s, no hump occurred. As shown in Fig. 3b, we applied NBS again to the TFT device where the hump was generated by PBS to observe how the hump changed. The hump gradually decreased as NBS continued. It completely disappeared after 10,000 s. Therefore, it was clearly found that the hump phenomenon in the a-IGZO TFTs occurred only by positive gate bias. Meanwhile, according to previous studies on reliabilities of a-IGZO TFTs, impact ionization by strong lateral electric field in drain region of a-IGZO semiconductors can cause hump^20,23,24^. The electrical reliability in terms of hump against high drain field stress in the a-IGZO TFT was then investigated by applying continuous DC stress under a drain voltage of + 25 V and a gate voltage of + 5 V (V_dg_ =  + 20 V) for 10,000 s. As shown in Fig. 4, there was only a slight increase in drain current after drain current stress. No hump phenomenon appeared. Therefore, the hump phenomenon in our a-IGZO TFTs was not related to the lateral electrical field induced by the high drain voltage. Instead, it was caused by the vertical electrical field formed by gate voltage.

### Characterization and analysis for hump generation in top gate a-IGZO TFTs

**Figure 5:**
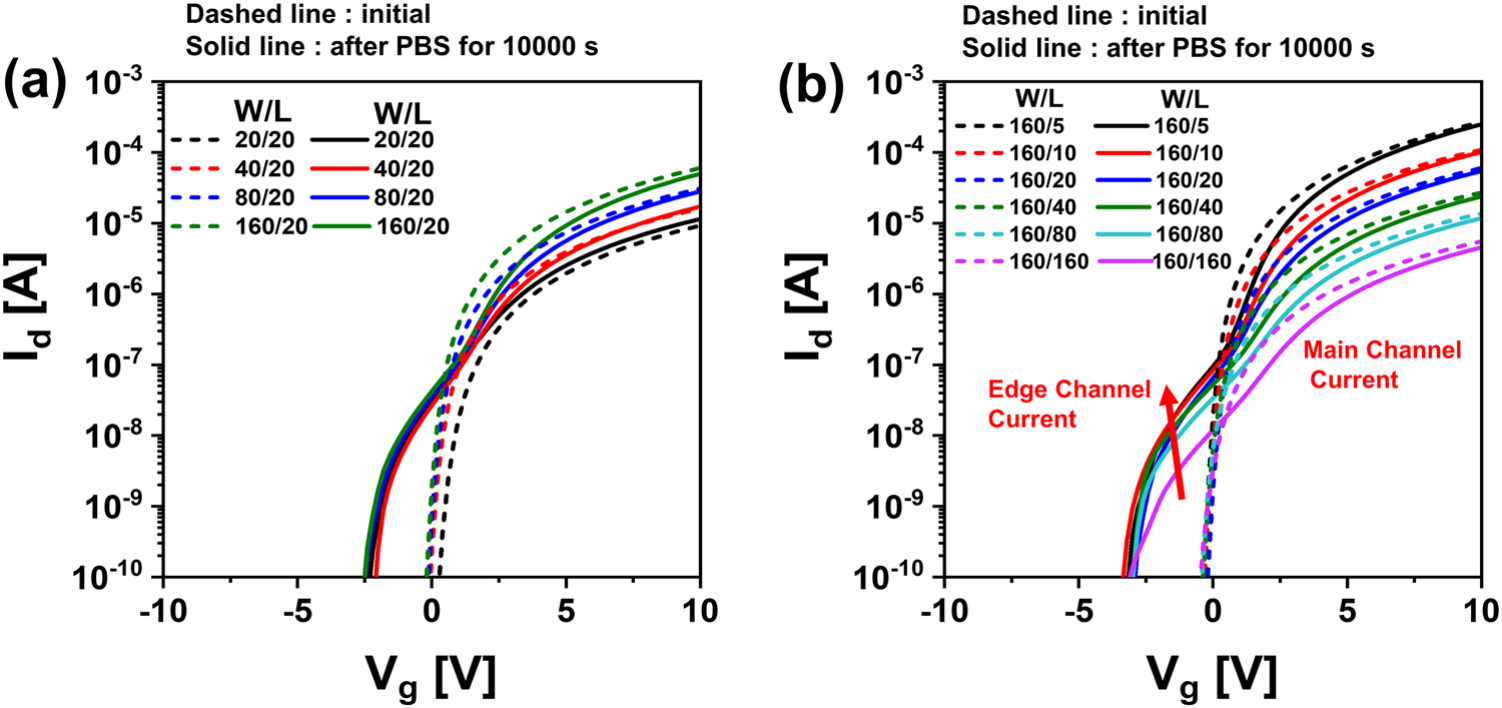
I–V characteristics for a-IGZO TFTs of initial states and after PBS of 10,000 s with (**a**) different channel widths and (**b**) different channel lengths.

Several studies have been conducted on hump due to a gate bias stress of a-IGZO TFTs^15–20^. Hump phenomena are known to occur when shallow donor species such as ionized vacancies, metal interstitials, and hydrogen interstitials in a-IGZO semiconductors are trapped in the channel interface or a-IGZO edge region, or when they are trapped in the back channel. We observed changes in I–V curves after applying PBS by 10,000 s to respective TFT devices with various channel widths and channel lengths to understand the generation mechanism of hump caused by gate bias applied to a-IGZO TFTs. Figure 5a shows initial I–V curves of a-IGZO TFTs with a channel length fixed at 20 μm and different channel widths of 20, 40, 80, and 160 μm and I–V curves of those after PBS of 10,000 s. Figure 5b shows I–V curves of IGZO TFTs with a channel width fixed at 160 μm and different channel lengths of 5, 10, 20, 40, 80, and 160 μm before and after PBS. Dashed lines are initial I–V characteristics of a-IGZO TFTs and solid lines are I–V characteristics of those after PBS 10,000 s. As shown in Fig. 5a, the size of the hump was irrelevant to the change in channel width of TFTs. On the other hand, as shown in Fig. 5b, the channel length had an obvious effect. The current level of the hump increased as the channel length was shortened. Considering that the hump phenomenon depended only on channel length, it suggested that a parallel type of parasitic transistor was created in the edge region of a-IGZO active island by continuous PBS, resulting in a hump. We thought that it would not be favorable to explain the gradual hump generation using a shallow donor model related to charged defects such as ionized vacancies, metal interstitials, or hydrogen interstitials created in a-IGZO by PBS. This is because shallow donors in the a-IGZO semiconductor can have more stable formation energy when the Fermi level (E_F_) is moved away from the conduction band edge by negative gate bias^25,26^. In other words, it is difficult to suggest that these shallow donors could directly affect the hump because they must be reduced within the IGZO bulk in PBS. In addition, the device characteristics were compared by varying barrier thicknesses such as 2 nm, 5 nm, and 50 nm to compare whether SiO_2_ serves as a diffusion blocking layer or is more effective than Al_2_O_3_ layer. Figure S1a–c show IGZO TFTs with Al_2_O_3_ barrier of 50 nm, 5 nm, and 2 nm, respectively. In Al_2_O_3_ barrier, hump occurred at 2 nm, but hump did not occur at 5 nm. On the other hand, Fig. S2a–c show IGZO TFTs with SiO_2_ barrier of 50 nm, 5 nm, and 2 nm, respectively. In SiO_2_ barrier, the size of hump decreased as the thickness increased, but hump still occurred even at 50 nm. Therefore, it was confirmed that Al2O3 ALD thin film acts as an excellent barrier for Na, but the SiO_2_ ALD thin film is difficult to use as a good barrier.

**Figure 6:**
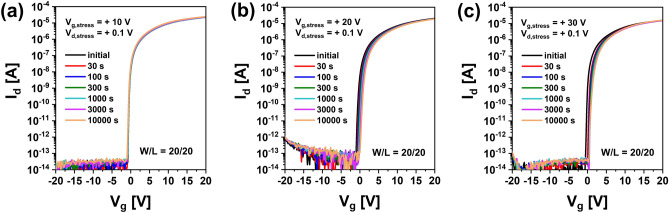
I–V characteristics of a-IGZO TFTs with Al_2_O_3_ buffer layer on glass substrate according to positive gate bias stress of (**a**) V_g_ =  + 10 V, (**b**) V_g_ =  + 20 V, and (**c**) V_g_ =  + 30 V.

On the other hand, this hump phenomenon might be caused by ionic chemical species that have moved from the glass substrate. To prevent ionic chemical species that could affect electrical properties of the TFT coming across from the glass substrate, an Al_2_O_3_ barrier layer (50 nm) was formed on top of the glass substrate using PEALD. All other processes were done under the same conditions to fabricate the a-IGZO TFT. Figure 6 shows I–V curves a-IGZO TFTs with Al_2_O_3_ barrier under PBS with gate voltage of + 10 V, + 20 V, and + 30 V, respectively. Interestingly, there was no hump in these TFTs at all. From the fact that Al_2_O_3_ barrier can solve this problem, the cause of the hump could be diffusion of some cation species to the a-IGZO layer from the glass substrate during a high temperature post-annealing of a-IGZO TFT.

**Figure 7:**
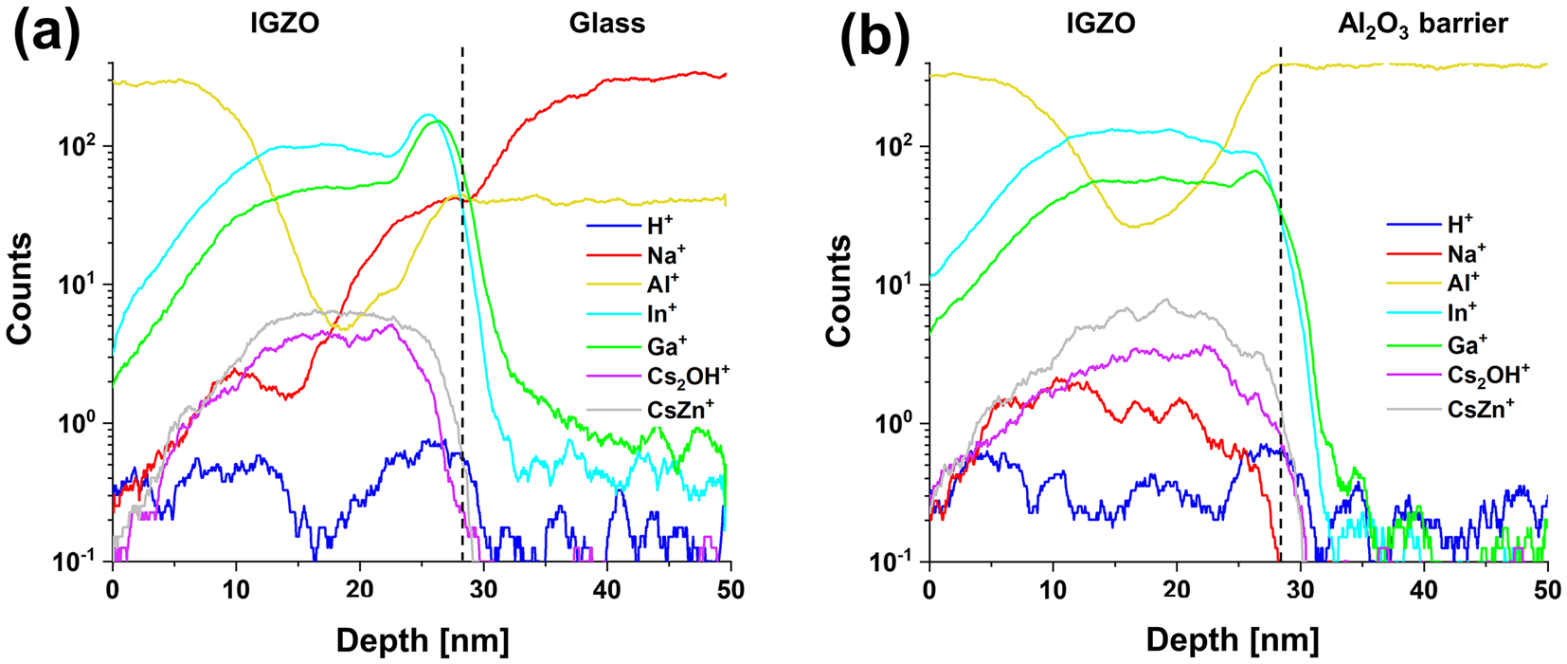
TOF–SIMS data of (**a**) an a-IGZO film without Al_2_O_3_ barrier layer and (**b**) an a-IGZO film with Al_2_O_3_ barrier layer.

**Figure 8:**
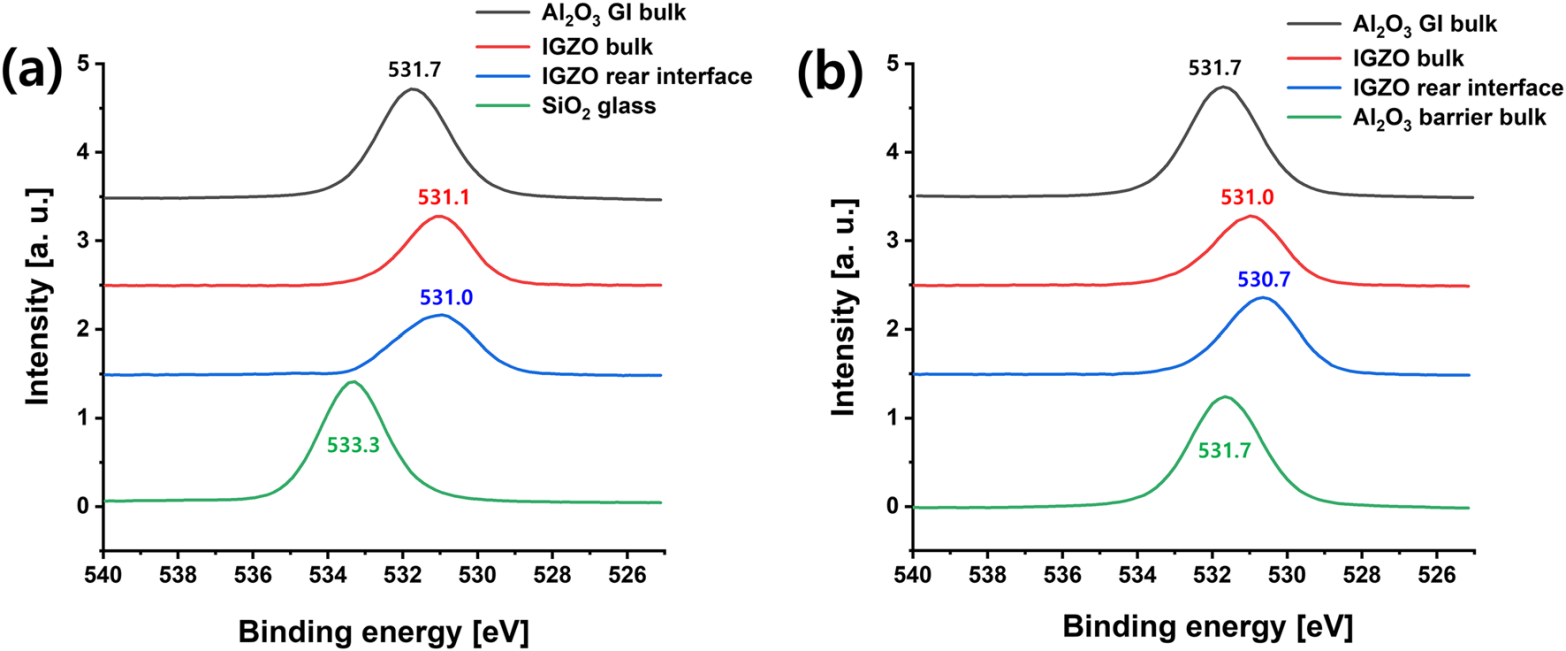
X-ray photoelectron spectroscopy O1s spectra of (**a**) Al_2_O_3_/a-IGZO/glass stack and (**b**) Al_2_O_3_/a-IGZO/ Al_2_O_3_/glass stack.

TOF–SIMS analysis was conducted to determine which ionic impurity diffused into the a-IGZO layer affected the hump. With respect to analysis targets, proton, hydroxyl ions, Na ions, and Al ions were considered as candidates of ion species that might affect electrical properties of TFTs by diffusion into IGZO from a glass substrate. Figure 7a shows TOF–SIMS data for a-IGZO thin film without Al_2_O_3_ barrier and Fig. 7b shows data for a-IGZO thin film with Al_2_O_3_ barrier. Comparing these two SIMS data, it was found that numbers of proton and hydroxyl ions were as small as being negligible (only a few counts for H and OH). Thus, their changes in IGZO according to the presence or absence of Al_2_O_3_ barrier might be insignificant, making it difficult to affect the hump in the a-IGZO TFT. It was also found that Na ions diffused a lot inside a-IGZO film when there was no Al_2_O_3_ barrier and that Al ions diffused a lot inside IGZO film when there was an Al_2_O_3_ barrier. Since the hump phenomenon was only found in a-IGZO TFTs without barrier layer, Al diffusion was not the cause of the hump. Thus, it could be concluded that Na ions should be the chemical species that caused the hump. In addition, XPS analysis was conducted to investigate the basic film quality of a-IGZO on a glass, a-IGZO on Al_2_O_3_, and Al_2_O_3_. Figure 8 shows O1s spectra of Al_2_O_3_/a-IGZO/glass stack and Al_2_O_3_/a-IGZO/Al_2_O_3_/glass stack. The binding energy of O1s peak of Al_2_O_3_ bulk layer was 531.7 eV in both stacks. a-IGZO (531.1 eV) on glass and a-IGZO (531.0 eV) on Al_2_O_3_ showed similar O1s spectra in the IGZO bulk film layers. However, the O1s spectrum was quite different in the region close to the rear interface of a-IGZO. a-IGZO (531.0 eV)/glass interface showed relatively higher binding energy than IGZO (530.7 eV)/Al_2_O_3_ interface. This showed that the chemical bonding environment of the IGZO rear interfaces might vary considerably due to the difference of inorganic layers such as SiO_2_ and Al_2_O_3_ despite the same binding energy in the a-IGZO bulk regions of the two stacks. Meanwhile, we fabricated a-IGZO TFTs using a Si wafer including SiO_2_ as an alkali-free substrate. Figure S3 shows the I–V characteristics of a-IGZOTFT on the Si wafer substrate according to the PBS time, but hump did not occur at all. Therefore, by using a Si wafer as an alkali-free substrate, it was possible to reconfirm that Na^+^ in the substrate was the cause of time-delayed hump in the I–V curve.

### Interpretation of hump generation mechanism in top gate a-IGZO TFTs

**Figure 9:**
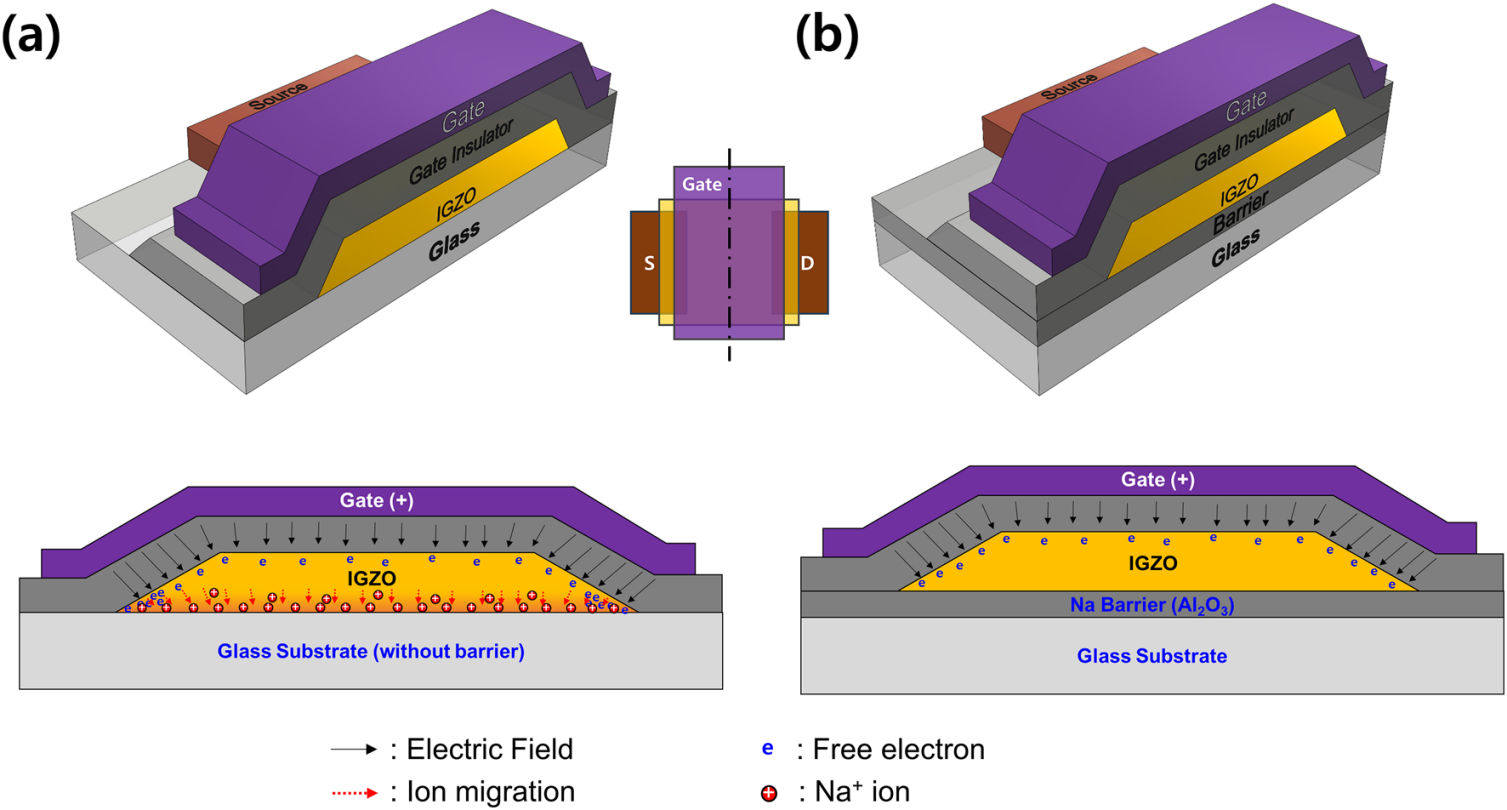
3D-device structures and cross-sectional views of the cross-section passing through the center of the gate in the plan view of the IGZO TFTs; (**a**) the TFTs without Al_2_O_3_ barrier and (**b**) the TFTs with Al_2_O_3_ barrier.

**Figure 10:**
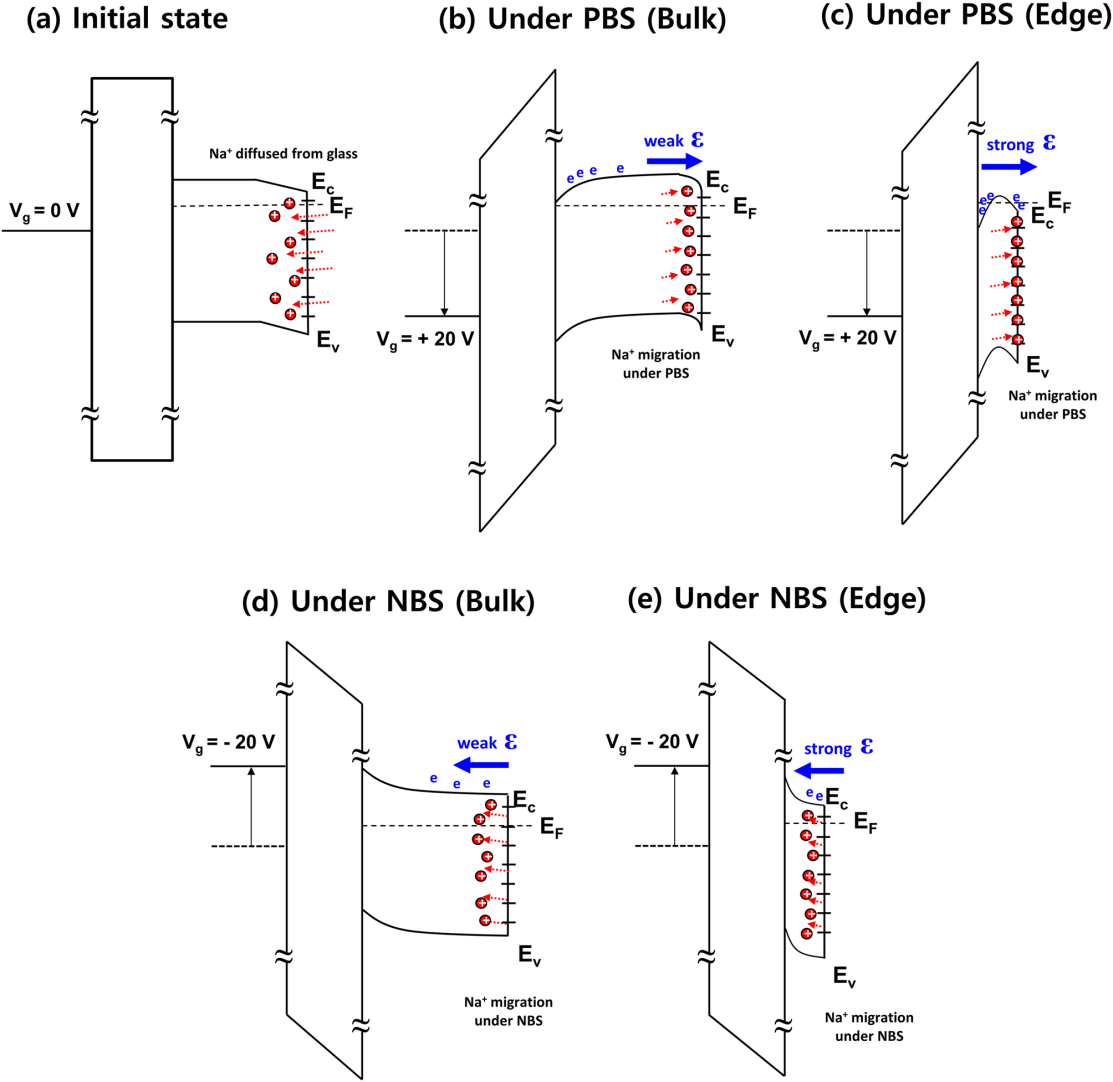
Energy band diagrams of a-IGZO TFTs (**a**) at initial state after high-temperature annealed, (**b**) at bulk region under long-term PBS condition, (**c**) for generation of parasitic transistors causing a hump on the edge region under long-term PBS condition (Na ions would be quickly trapped, and conduction band lowering might occur first at the edge region.), (**d**) at bulk region under long-term NBS condition, and (**e**) on the edge region under long-term NBS condition (The trapped Na ions would be de-trapped from the IGZO interface and migrated in the direction of the front channel.).

We examined how Na ions diffused into IGZO could cause a hump. Figure 9 shows 3D-device structures and cross-sectional views of the cross-section passing through the center of the gate in the plan view of the IGZO TFTs. Figure 9a,b show the TFTs without Al_2_O_3_ barrier and with Al_2_O_3_ barrier, respectively. Especially, Fig. 9a illustrated the process of creating parasitic transistors on edge regions of a-IGZO active island that could cause a hump. To understand the origin of the hump characteristics, TOF–SIMS analysis data before and after post-annealing of 350 °C were compared as shown in Fig. S4. Although post-annealing obviously increased the diffusion of Na ions, Na ions were identified within a-IGZO on glass even before post-annealing. It is believed that the diffused Na ions were detected in a-IGZO even before annealing due to a heat treatment effect by the high temperature deposition process (250 °C) of the Al_2_O_3_ gate insulator. Therefore, rather than Na + diffusion being the direct cause of hump, Na + migration (drift) due to an electric field by gate bias might be the cause of hump. Na^+^ could be migrated to the interface of glass and a-IGZO due to a vertical electrical field formed under PBS. Figure 10 shows an energy band diagram to explain the mechanism of hump generation due to Na^+^ diffused in the IGZO film by the gate electric field. Figure 10a shows an initial flat-band state of a-IGZO TFT during high-temperature annealing. Due to diffused Na ions from the glass substrate, the energy band would be slightly bent behind the a-IGZO layer. Figure 10b,c show a-IGZO band bending by Na^+^ migration into the back of the IGZO by the electric field under long-term PBS, presenting the mechanism of hump generation accordingly. Regarding edge regions, both sidewalls of the IGZO are thin. The electric field on the IGZO edge regions would be stronger than that on the a-IGZO bulk layer. Na ions would be quickly trapped, and conduction band lowering might occur first at the edge region. Eventually, the channel of parallel transistors created in the edge regions might open early, causing a hump. In other words, even if the same gate voltage was applied, a-IGZO on the edge region might reach an accumulation state first. IGZO channel on the edge region could be formed earlier than that on the bulk region, leading to the occurrence of a hump. On the other hand, as shown in Fig. 10d,e, when NBS was applied, the trapped Na ions would be de-trapped from the IGZO interface and migrated in the direction of the front channel. Therefore, electrons accumulated at the edge would be depleted and the hump disappeared.

## Conclusion

Top gate a-IGZO TFTs post-annealed at high temperature of 350 °C showed good field effect mobility with uniform V_th_ and SS at their I–V characteristics according to different channel widths and lengths. However, hump occurred in the subthreshold region when they were exposed to positive gate DC bias stress for a long time (more than 3600 s). This stress-induced hump accelerated at higher positive gate voltage but disappeared by negative gate voltage. In particular, the current level of the hump increased as the channel length was shortened, while it was independent on channel width. It was thought that parallel parasitic transistors might have been created in the edge region of a-IGZO active island during sustained PBS. This could be caused by ionic chemical species moving from the glass substrate. TOF-SIMS analysis confirmed that Na ions were significantly diffused into a-IGZO from glass substrate where there was no Al_2_O_3_ barrier layer. To prevent ionic chemical species that could affect electrical properties of the TFT from coming across from the glass substrate, an Al_2_O_3_ barrier layer was formed between a-IGZO TFT and glass substrate, leading to no hump in the resultant TFT at all. Mobile Na ions might have migrated to the edge and back side of a-IGZO active island by a vertical gate electric field. Parasitic transistors created at the edge regions might have been turned on earlier to generate hump. Therefore, when Al_2_O_3_ layer was formed under a-IGZO TFT, this hump phenomenon could certainly be solved by working as a barrier against Na ions coming cross into a-IGZO.

### Supplementary Information

Supplementary Figures.

## Data Availability

The data that support the findings of this study are available within the article.
