# Supplementary material for: Investigating an abnormal hump phenomenon in top gate a-InGaZnO thin-film transistors due to mobile sodium diffusion
Source: Sci Rep. 2023 Aug 22;13:13714. doi: 10.1038/s41598-023-40664-x (PMC10444848; doi:10.1038/s41598-023-40664-x)
Supplement: Supplementary file 1 — Supplementary Figures. [file 41598_2023_40664_MOESM1_ESM.docx]

**Supplementary Information**

**Investigating an abnormal hump phenomenon in top gate a-InGaZnO thin-film transistors due to mobile sodium diffusion**

So Hee Park^1^, Min Young Kim^1^, Hyeong Wook Kim^1^, Changyong Oh^1,2^, Hyeong Keun Lee^3^ & Bo Sung Kim^1,2,3,^*

^1^Department of Applied Physics, Korea University, Sejong, 30019, Republic of Korea

^2^E·ICT-Culture·Sports Track, Korea University, Sejong, 30019, Republic of Korea

^3^Division of Display and Semiconductor Physics, Korea University, Sejong, 30019, Republic of Korea

^*^Corresponding author, E-mail address : bskim86@korea.ac.kr


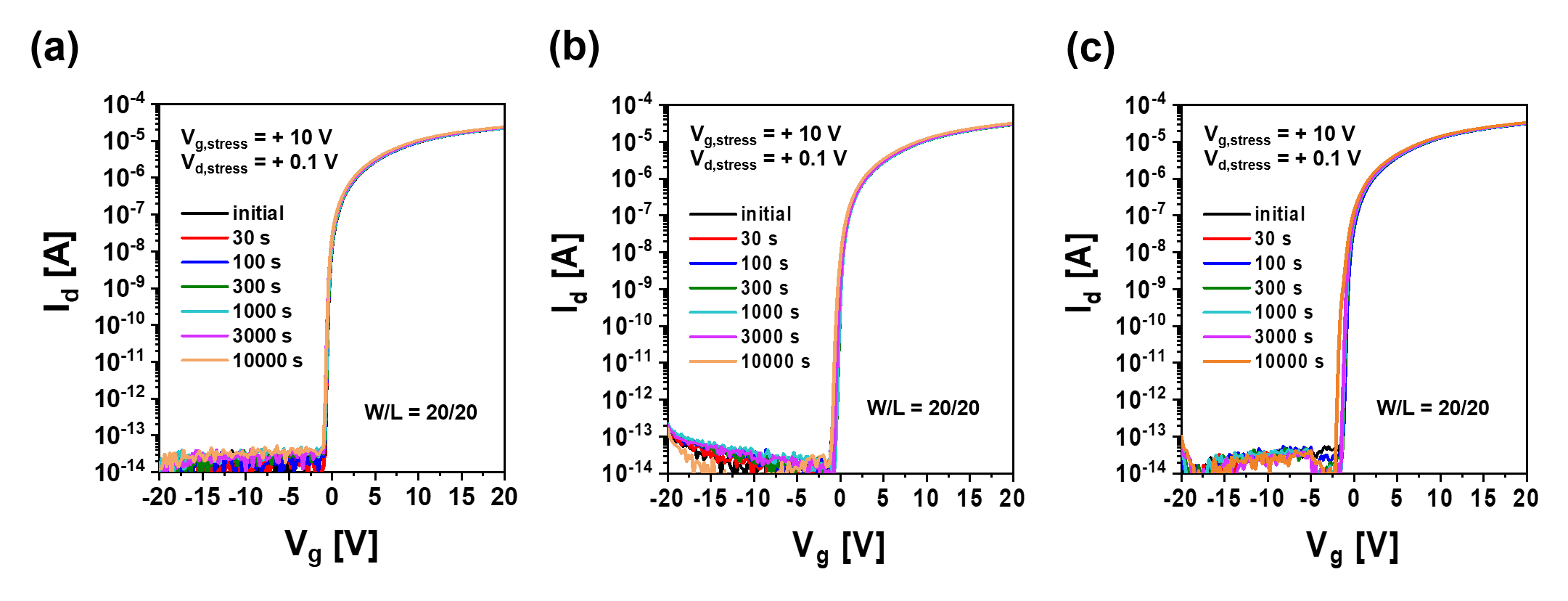


Fig. S1. I-V characteristics of a-IGZO TFTs with Al_2_O_3_ barrier layer of (a) 50 nm, (b) 5 nm, and (c) 2 nm on glass substrate as a function of positive gate bias stress time (V_g,stress_ = +10 V, V_d,stress_ = +0.1 V).


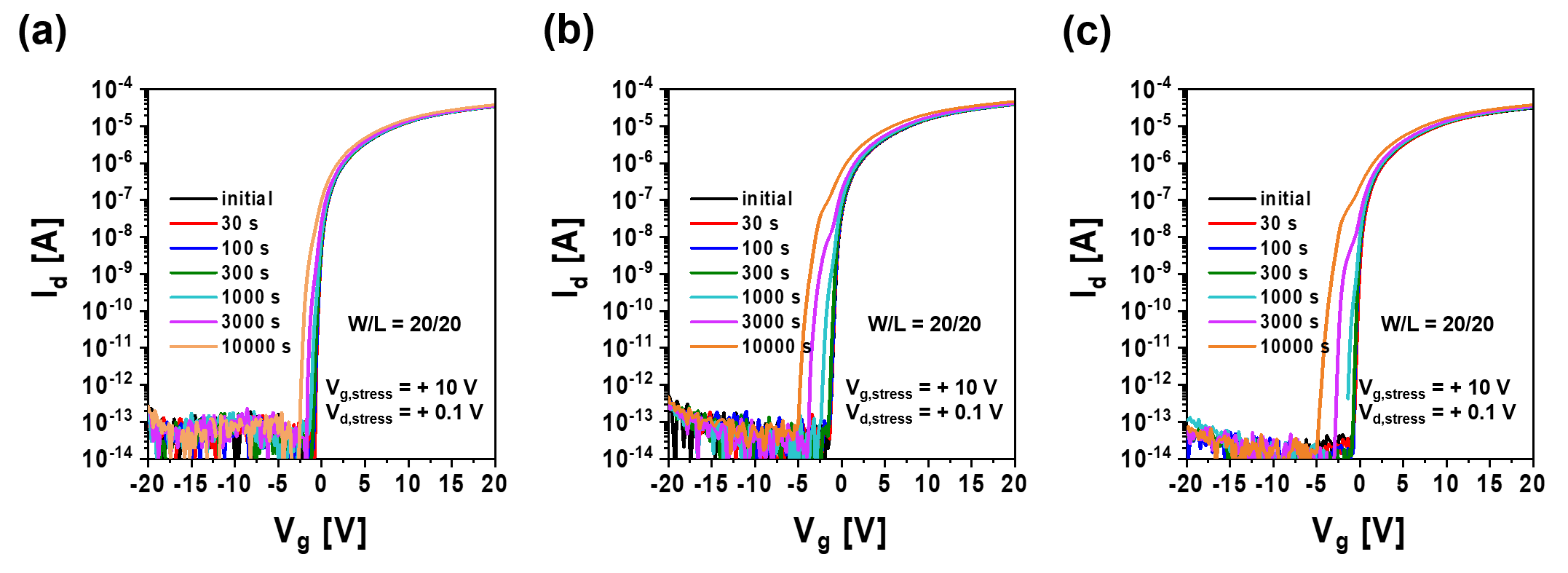


Fig. S2. I-V characteristics of a-IGZO TFTs with SiO_2_ barrier layer of (a) 50 nm, (b) 5 nm, and (c) 2 nm on glass substrate as a function of positive gate bias stress time time (V_g,stress_ = +10 V, V_d,stress_ = +0.1 V).


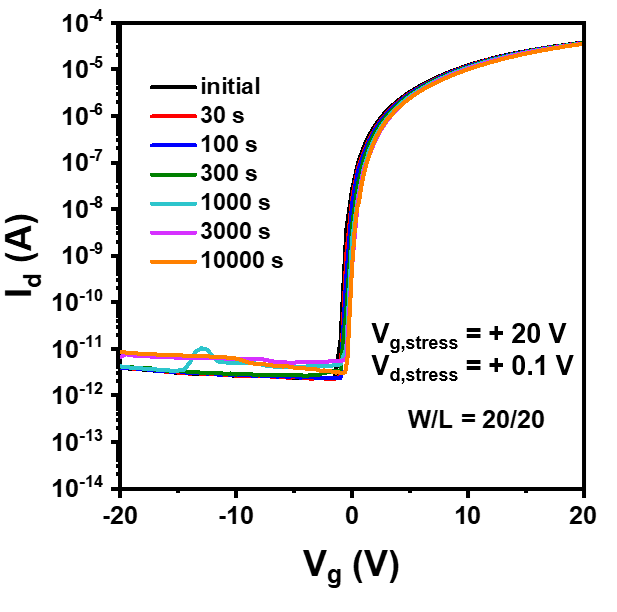


Fig. S3. I-V characteristics of a-IGZOTFT on a Si wafer substrate as a function of positive gate bias stress time time (V_g,stress_ = +20 V, V_d,stress_ = +0.1 V).


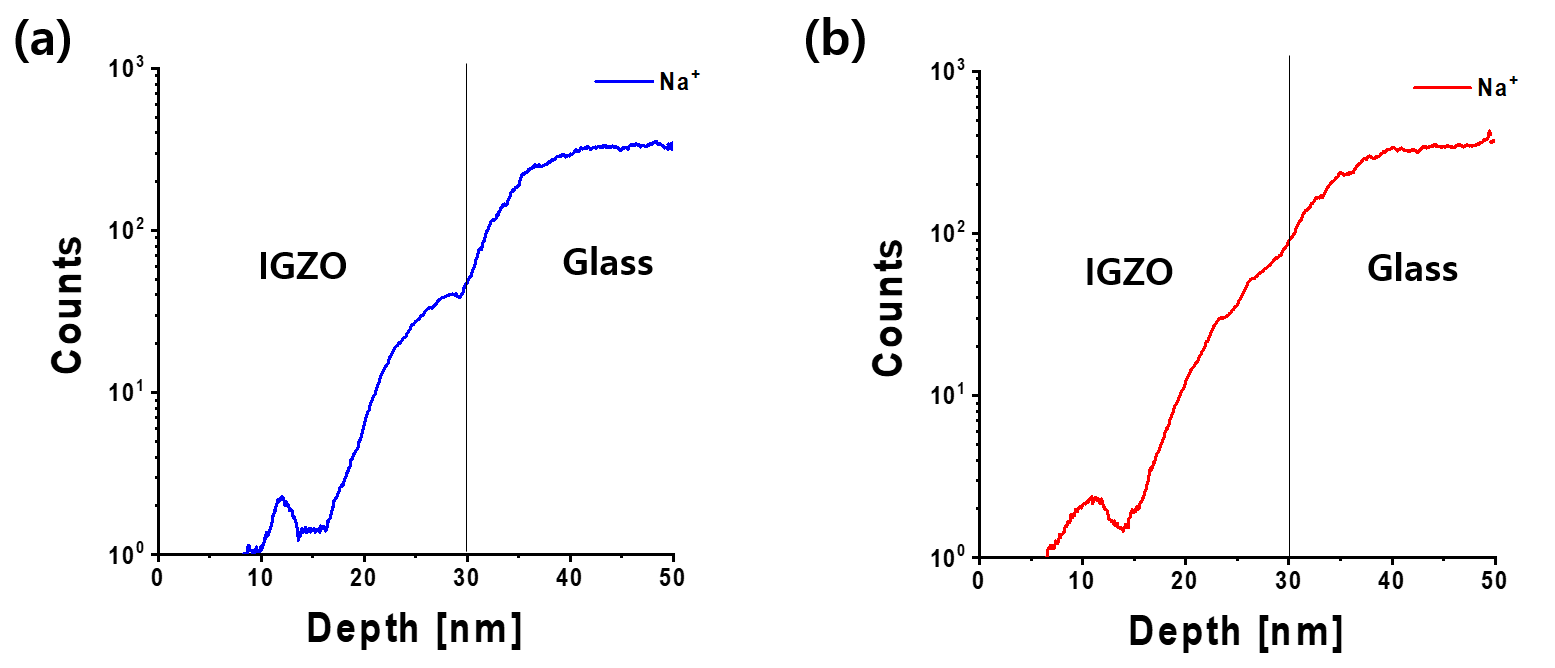


Fig. S4. TOF-SIMS analysis data of IGZO on glass substrate (a) before and (b) after post-annealing of 350 °C.
